# Erratum for Nunes et al., “Recurrent *Campylobacter jejuni* Infections with *In Vivo* Selection of Resistance to Macrolides and Carbapenems: Molecular Characterization of Resistance Determinants”

**DOI:** 10.1128/spectrum.03121-23

**Published:** 2023-10-10

**Authors:** Alexandra Nunes, Mónica Oleastro, Frederico Alves, Nadia Liassine, David M. Lowe, Lucie Benejat, Astrid Ducounau, Quentin Jehanne, Vítor Borges, João Paulo Gomes, Gauri Godbole, Philippe Lehours

## ERRATUM

Volume 11, no. 4, e01070-23, 2023, https://doi.org/10.1128/spectrum.01070-23. Page 1, byline: Philippe Lehours’ name should appear as shown in this Erratum.

