# Supplementary material for: Recurrent Campylobacter jejuni Infections with In Vivo Selection of Resistance to Macrolides and Carbapenems: Molecular Characterization of Resistance Determinants
Source: Microbiol Spectr. 2023 Jun 26;11(4):e01070-23. doi: 10.1128/spectrum.01070-23 (PMC10434052; doi:10.1128/spectrum.01070-23)
Supplement: Supplemental file 1 — Supplemental material. Download spectrum.01070-23-s0001.docx, DOCX file, 0.03 MB [file spectrum.01070-23-s0001.docx]

**Supplementary material**

**Materials and Methods**

*Bacterial culture*

Upon receipt, the isolates were subcultured under microaerobic atmosphere (6% O2, 5-10% CO2, 80-90% N2, 5-10% H2) in jars using an Anoxomat microprocessor (Mart Microbiology, B.V. Lichtenvoorde, The Netherlands). Species identification was confirmed by MALDI-TOF mass spectrometry (Bruker Daltonics, Bremen, Germany) (1). All strains were conserved at -80°C in homemade brucella broth supplemented with 20% glycerol.

*Antimicrobial susceptibility testing – Agar dilution*

For ertapenem and meropenem, MH-F agar plates were prepared with or without each carbapenem to be tested. For each carbapenem, MIC was determined by two independent readers as the lowest drug concentration inhibiting visible growth of each isolate. The reference strain *C. jejuni* ATCC 33560 was used as a quality control strain. A stock solution of 100 mg/mL of each compound (Ertapenem, INVANZ 1g, MSD – Lot R013489) (Meropenem, KABI 1g, Fresenius – Lot MFR1018) was prepared in sterile water. Then adapted dilutions were prepared from 25,600 mg/L to 400 mg/L in order to obtain agar plates containing a range of concentrations from 512 mg/L to 1 mg/L. The inoculation was performed with a multiple inoculator derived from a Steers' apparatus. The plates were incubated for 24h at 35°C in a workstation under a microaerobic atmosphere (5% O2, 10% CO2, 85% N2).

*Extraction of total DNA*

For all isolates from clinical Case A, total DNA was extracted from bacterial lysates using the MagNA Pure 6 DNA and Viral NA SV Kit on a MagNA Pure 96 System (Roche Applied Science). For Case B, DNA was extracted on the NucliSens easyMAG platform (bioMérieux). Quantification and purity checks were determined by spectrophotometry (NanoDrop Technologies).

*Genome assembly*

After reads’ quality analysis using FastQC v0.11.5 (http://www.bioinformatics.babraham.ac.uk/projects/fastqc/) and cleaning with Trimmomatic v0.38 (http://www.usadellab.org/cms/?page=trimmomatic) (2), genomes were de novo assembled with SPAdes 3.14.0 (http://bioinf.spbau.ru/spades) (3) with a mean depth of coverage above 160x, and subsequently improved using Pilon v1.23 (4).

*Analysis of accessory genome*

The obtained draft genome sequences were analysed using PHASTER (<http://phaster.ca/>) to detect phages, as well as MobileElementFinder (<https://cge.food.dtu.dk/services/MobileElementFinder/>) to detect mobile genetic elements (MGEs). The existence of putative plasmids was inspected by checking contigs >1,000 bp with a high depth of coverage and evidence of circularity, while the presence of known plasmids was examined by reference-based mapping using Snippy v4.6.0 (https://github.com/tseemann/snippy) against an in-house list of plasmids already described for *Campylobacter* spp.

*Microevolutionary analysis*

In order to maximize the number of sites available for SNP and indel comparison and potentially provide greater discrimination resolution, trimmed reads were mapped against the draft genome of the first isolate collected from each clinical case with a susceptible phenotype for carbapenems (A1 and B1 isolates were used as reference genomes). Variants were called in sites with minimum mapping quality of 60, minimum base quality of 20, and minimum number of reads covering the variant position ≥ of 10. For each clinical case, all inter- and intra-strain SNPs and indels acquired throughout the microevolution of *C. jejuni* during infection were carefully inspected and confirmed using IGV v2.15.2 (http://software.broadinstitute.org/software/igv/) (5).

For each clinical case, an analysis of intra-strain in-length variability of homopolymeric (polyN) tracts was also conducted, considering a customized list of 41 polyN regions (Table S1) that: *i)* have already been identified to mediate phase variation in *C. jejuni* (6–9), or *ii)* are putative polyN tracts identified from our microevolutionary analysis. Briefly, for each polyN tract, an *in-house* script (https://github.com/insapathogenomics/countDNABox) was used to count, for each isolate, the exact number of reads carrying a specific polyN length, allowing the determination of the precise relative frequency of same-population clones carrying distinct base counts. Only nucleotide tracts with seven or more Gs or Cs as well as with 10 or more As or Ts were counted (6).

**Results**

*Microevolutionary analysis*

The microevolutionary analysis revealed the emergence of 23 SNPs (two thirds non-synonymous) and nine small indels among isolates from Case A (Table S3), affecting 25 loci including six promoter regions. Overall, six mutations resulted in smaller-sized proteins due to stop codons prematurely gained exclusively at three loci (Cj0834c, Cj1192 and Cj0125c NCTC 11168 homologues), differentially present in A3, A6 and A7 isolates, but the opposite situation (stop loss) was also seen to restore the full length of a malate oxidoreductase ORF (Cj1287c homologue) in all bacterial populations evolved from A1. For isolates from Case B, a total of five SNPs (four non-synonymous) and four small indels were found to affect either genetic diversity or gene expression of eight loci (Table S4). Two frameshifts were found to be B4-exclusive, leading to a 142bp shorter VirK virulence factor (essential for the antimicrobial peptide resistance (9) gene) as well as to two smaller ORFs both coding for ankyrin repeat-containing possible periplasmic proteins (in contrast to the intact Cj0834c homologue). For both clinical cases, most of the observed genetic diversity falls within genes or promoter regions of genes involved in antibiotic resistance, cell envelope and signal transduction. However, for Case A, loci associated with cell processes like chemotaxis and motility or transport, as well as to small molecule metabolism (e.g., fatty acid biosynthesis or energy) were also found to be differentially affected among isolates. Interestingly, half of the mutations found in these later categories (both in coding and promoting regions) were shared by all non-A1 isolates, reflecting their importance in *in vivo* bacterial adaptation to the immunocompromised host.

In-length variable polyN tracts (the majority poly-G/C) were additionally observed for 18 and 23 regions, inter- and intra-isolates from Case A and Case B, respectively (Table S5 and Figure S1). Of these, a total of 25 polyN regions (n=10 for Case A and n=15 for Case B) were found to display high genetic heterogeneity among isolates (Table S5), mostly in coding regions, potentially resulting in a reversible “ON/OFF” switching in gene expression and consequently on the status of the produced protein. As expected, most of the identified polyN regions affect transferases or enzymes with roles in biosynthesis and modification of bacterial lipopolysaccharide, capsule and flagella. For Case A, it was not possible to correlate the phasotype (i.e., frequency of in-length variable polyN tracts) diversity seen among isolates throughout the infection (Figure S1). For instance, the carbonic anhydrase gene (*maf7*) exhibited an enriched “ON” phasotype for both A1 and A5 (due to the presence of a 9-G/C string), but an “OFF” expression state for the major populations of A2 (due to an 11-G/C string) and A4/A7 (both due to a 10-G/C string). In contrast, for Case B, some polyN regions (like the Cj1295 homolog, *maf7*, *traG*, peg.280_B1) exhibited main allele frequencies that clearly differentiate B1 from all non-B1 isolates, resulting in distinct “ON”/“OFF” expression status (Figure S1). In addition, for the Cj1296 homolog, the isolates from feces (B3 and B5) displayed an enriched “OFF” phasotype that contrasts with the main “ON” expression state of the bacterial populations isolated in blood (B1, B2 and B4), for which the presence of a 10-G/C string allows translation to continue into Cj1297 homolog. Interestingly, for some polyN regions (Figure S1), the exhibited main phasotype was found to be conserved within Case B but variable among isolates from Case A (e.g., Cj0617 and Cj0031 homologs), and the contrary was also seen (e.g., for the Cj1295 homolog), suggesting a role in the adaptability to the respective immunocompromised host. On the other hand, for six phase-variable genes (Cj0685c, Cj1420c, Cj1305c_Cj1306c_Cj1310c and Cj1318 homologs), all isolates of both clinical cases expressed a homogeneous main phasotype, suggesting their importance during human colonization (9).

In conclusion, several mutations, phase variable or not, observed inter- or intra-isolates of both clinical cases were found to be likely important for either the adaptability to the immunocompromised host or during human infection/colonization. In agreement with this, all isolates harbored a functional version of the *cipA* gene, which was previously associated with *C. jejuni* persistent human infection (10).

**References**

1. Bessède E, Solecki O, Sifré E, Labadi L, Mégraud F. 2011. Identification of Campylobacter species and related organisms by matrix assisted laser desorption ionization-time of flight (MALDI-TOF) mass spectrometry. Clin Microbiol Infect 17:1735–1739.

2. Bolger AM, Lohse M, Usadel B. 2014. Trimmomatic: A flexible trimmer for Illumina sequence data. Bioinformatics 30:2114–2120.

3. Bankevich A, Nurk S, Antipov D, Gurevich AA, Dvorkin M, Kulikov AS, Lesin VM, Nikolenko SI, Pham S, Prjibelski AD, Pyshkin A V., Sirotkin A V., Vyahhi N, Tesler G, Alekseyev MA, Pevzner PA. 2012. SPAdes: A new genome assembly algorithm and its applications to single-cell sequencing. J Comput Biol 19:455–477.

4. Walker BJ, Abeel T, Shea T, Priest M, Abouelliel A, Sakthikumar S, Cuomo CA, Zeng Q, Wortman J, Young SK, Earl AM. 2014. Pilon: An integrated tool for comprehensive microbial variant detection and genome assembly improvement. PLoS One 9.

5. Robinson JT, Thorvaldsdóttir H, Winckler W, Guttman M, Lander ES, Getz G, Mesirov JP. 2011. Integrative Genome Viewer. Nat Biotechnol 29:24–6.

6. Aidley J, Wanford JJ, Green LR, Sheppard SK, Bayliss CD. 2018. Phasome It : an ‘ omics ’ approach to cataloguing the potential breadth of phase variation in the genus Campylobacter https://doi.org/10.1099/mgen.0.000228.

7. Burnham PM, Hendrixson DR. 2018. Campylobacter jejuni: collective components promoting a successful enteric lifestyle. Nat Rev Microbiol 16:551–565.

8. Kivistö RI, Kovanen S, Skarp-de Haan A, Schott T, Rahkio M, Rossi M, Hänninen M-L. 2014. Evolution and comparative genomics of Campylobacter jejuni ST-677 clonal complex. Genome Biol Evol 6:2424–2438.

9. Cayrou C, Barratt NA, Ketley JM, Bayliss CD. 2021. Phase Variation During Host Colonization and Invasion by Campylobacter jejuni and Other Campylobacter Species. Front Microbiol 12:1–11.

10. Crofts AA, Poly FM, Ewing CP, Kuroiwa JM, Rimmer JE, Harro C, Sack D, Talaat KR, Porter CK, Gutierrez RL, DeNearing B, Brubaker J, Laird RM, Maue AC, Jaep K, Alcala A, Tribble DR, Riddle MS, Ramakrishnan A, McCoy AJ, Davies BW, Guerry P TM. 2018. Campylobacter jejuni transcriptional and genetic adaptation during human infection. Nat Microbiol 3:496–502.
