# Supplementary figures and images for: Recurrent Campylobacter jejuni Infections with In Vivo Selection of Resistance to Macrolides and Carbapenems: Molecular Characterization of Resistance Determinants
Source: Microbiol Spectr. 2023 Jun 26;11(4):e01070-23. doi: 10.1128/spectrum.01070-23 (PMC10434052; doi:10.1128/spectrum.01070-23)

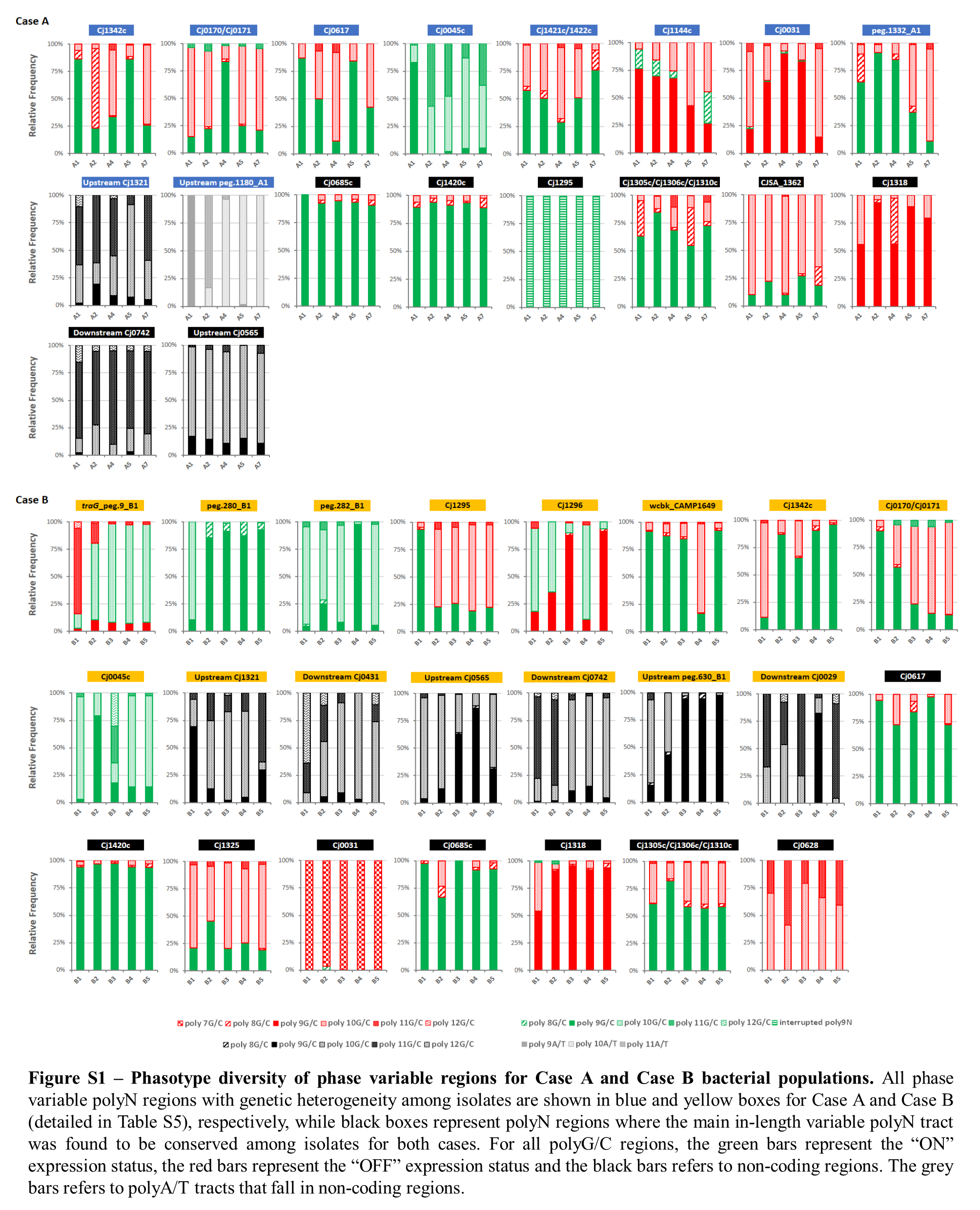

Supplement: Supplemental file 2 — Figure S1. Download spectrum.01070-23-s0002.tif, TIF file, 2.3 MB [file spectrum.01070-23-s0002.tif]
