# Supplementary material for: Recurrent Campylobacter jejuni Infections with In Vivo Selection of Resistance to Macrolides and Carbapenems: Molecular Characterization of Resistance Determinants
Source: Microbiol Spectr. 2023 Jun 26;11(4):e01070-23. doi: 10.1128/spectrum.01070-23 (PMC10434052; doi:10.1128/spectrum.01070-23)
Supplement: Supplemental file 4 — Table S2. Download spectrum.01070-23-s0004.docx, DOCX file, 0.01 MB [file spectrum.01070-23-s0004.docx]

**Supplementary Table S2 - European Nucleotide Archive (ENA) accession information.**

| **Isolate ID** | **BioProject ID** | **Sample ID** | **Isolate Alias** |
| --- | --- | --- | --- |
| Cje_A1 | PRJEB42628 | ERS5597190 | Cje_A1 |
| Cje_A2 | PRJEB42628 | ERS5597191 | Cje_A2 |
| Cje_A4 | PRJEB42628 | ERS5597192 | Cje_A4 |
| Cje_A5 | PRJEB42628 | ERS5597193 | Cje_A5 |
| Cje_A7 | PRJEB42628 | ERS5597194 | Cje_A7 |
| Cje_B1 | PRJNA505131 | SRS5681549 | 301854 |
| Cje_B2 | PRJNA505131 | SRS5302100 | 318281 |
| Cje_B3 | PRJNA505131 | SRS5305034 | 320281 |
| Cje_B4 | PRJNA505131 | SRS5299442 | 325507 |
| Cje_B5 | PRJNA505131 | SRS5301507 | 328550 |
